# Quantitative Parameters Derived Using the Biexponential and Stretched Exponential Models for the Detection of Early Renal Impairment in Chronic Kidney Disease

**DOI:** 10.2174/0115734056445507251111061849

**Published:** 2025-11-24

**Authors:** Yi Dai, Zhucheng Lu, Yidi Chen, Keqiang Huang, Zhenyuan Xia, Lan Lan, Wei Li, Haiyuan Wei, Xuejie Yang, Xiamei Chen, Liling Long, Wenzhao Yuan

**Affiliations:** 1 Department of Radiology, The First Affiliated Hospital of Guangxi Medical University, No. 6 Shuangyong Road, Nanning, Guangxi - 530021, China; 2 Department of Radiology, The Second Affiliated Hospital of Guangxi Medical University, No. 166 Daxuedong Road, Nanning, Guangxi - 530007, China; 3 Department of Radiology, Liuzhou Traditional Chinese Medical Hospital, No. 32 Jiefang North Road, Liuzhou, Guangxi - 545001, China; 4 Department of Pathology, The First Affiliated Hospital of Guangxi University of Chinese Medicine, No. 89-9 Dongge Road, Nanning, Guangxi - 530023, China; 5 Department of Nephrology, The Second Affiliated Hospital of Guangxi Medical University, No. 166 Daxuedong Road, Nanning, Guangxi - 530007, China; 6 Guangxi University of Chinese Medicine, No. 13 Wuhe Avenue, Nanning, Guangxi - 530200, China; 7 MR Application, GE Healthcare Co., Ltd., Beijing - 100176, China

**Keywords:** Magnetic resonance imaging, Biexponential model, Stretched exponential model, Chronic kidney disease, Pathology, Renal Impairment

## Abstract

**Introduction:**

The biexponential model of Intravoxel Incoherent Motion (IVIM) has been applied to estimate renal damage. However, the role of the biexponential and stretched exponential models in assessing early renal damage in Chronic Kidney Disease (CKD) is unclear.

**Methods:**

In this prospective study, 61 patients with CKD and 19 healthy volunteers underwent renal IVIM imaging. The monoexponential model yielded the Apparent Diffusion Coefficient (ADC); the biexponential model provided the true diffusion coefficient (ADC_slow_), pseudo-diffusion coefficient (ADC_fast_), and perfusion fraction (*f*); and the stretched-exponential model provided the Distributed Diffusion Coefficient (DDC) and diffusion heterogeneity index alpha (*α*). The estimated Glomerular Filtration Rate (eGFR) was calculated for all participants, and pathological scores were assessed in CKD patients. Correlations of ADC, ADC_slow_, ADC_fast_, *f*, DDC, and *α* with eGFR and pathological scores were analyzed. Receiver operating characteristic analysis compared the diagnostic performance of ADC_slow_, ADC_fast_, *f*, DDC, and *α* for grading renal pathological injury.

**Results:**

ADC_fast_, *f*, and *α* showed high diagnostic accuracy in differentiating controls from CKD patients (AUC: 0.964, 0.974, and 0.981, respectively), as well as from CKD patients with high eGFR (AUC: 0.933, 0.952, and 0.966, respectively). Pathological scores were significantly higher in the low eGFR group than in the high eGFR group (*P* < 0.05). ADC_fast_, *f*, and *α* were negatively correlated with pathological scores (*P* < 0.05).

**Discussion:**

Renal cortical ADC_fast_, *f*, and *α* are sensitive biomarkers of early renal injury in CKD even when eGFR is preserved. Moreover, the ADC_fast_ and *f* values of the renal cortex were significantly correlated with tubulointerstitial damage. The primary limitations of this study are the single-center data and the limited scope of the region of interest. Further work is needed to recruit more participants, and those results will be verified by external centers.

**Conclusion:**

Biexponential and stretched exponential model-derived parameters may be superior to monoexponential model-derived parameters for evaluating early renal damage in CKD.

## 1 INTRODUCTION

The incidence rate of Chronic Kidney Disease (CKD) remains significantly high [1], particularly among inpatients with diseases such as diabetes and hypertension, where it reaches as high as 17.8% [2]. The Kidney Disease: Improving Global Outcomes (KDIGO) guidelines emphasize the role of estimated Glomerular Filtration Rate (eGFR) in assessing CKD progression, but note its limitations for analyzing early renal dysfunction, particularly in stage I CKD, where the eGFR is influenced by diet and metabolism. Furthermore, the eGFR reflects average renal function over time, rather than real-time unilateral kidney status [3, 4]. The KDIGO 2024 guidelines state that renal biopsy is the gold standard for the diagnosis of glomerular diseases; however, its invasive nature and associated risks, like hemorrhage and infection, make it unsuitable for long-term monitoring of renal lesions [5-7]. Therefore, more specific and non-invasive methods for estimating renal function and monitoring lesions in CKD are needed.

Magnetic Resonance Imaging (MRI) is increasingly being used for the investigation of renal conditions such as clear cell renal cell carcinoma, kidney transplantation, and CKD [8-10]. The biexponential model of Intravoxel Incoherent Motion (IVIM) can be used to simultaneously estimate microvascular perfusion and water-molecule diffusion by means of the quantitative parameters pseudo-diffusion coefficient (ADC_fast_), true diffusion coefficient (ADC_slow_), and perfusion fraction (*f*) [11, 12]. These parameters differ significantly in patients with Immunoglobulin A (IgA) nephropathy-induced renal damage and in control subjects [13]. Additionally, significant differences in ADC_slow_, *f*, and ADC_fast_ values have been found between healthy volunteers and CKD patients with normal (CKD stage G1-G2) and abnormal eGFR (CKD stage G3-G5) [14, 15]. Furthermore, IVIM parameters (e.g., cortical ADC_fast_ values) have been proposed as an index for differentiating diabetic nephropathy in clinical practice [16].

In theory, the biexponential model of IVIM may accurately evaluate renal function and microstructural changes; however, it does not account for the continuous distribution of intravoxel diffusion coefficients. Therefore, Park *et al*. suggested using alpha (*α*) and Distributed Diffusion Coefficient (DDC) from the stretched exponential model to evaluate intravoxel diffusion heterogeneity and distributed diffusion effect, respectively [17]. Yuan *et al*. recruited CKD patients and healthy controls who underwent multi-b-value diffusion sequence scans; the results suggested that a combined model of cortical *f,* Mean Kurtosis, and eGFR provides a useful comprehensive tool for grading interstitial fibrosis, with cortical *f* and α values serving as potential biomarkers for CKD progression [18].

Thus far, few prospective studies have investigated the value of stretched exponential models for the evaluation of CKD-related pathological renal damage, particularly in the early stages. This study aims to clarify the ability of parameters derived using the biexponential and stretched exponential models to detect early renal damage in CKD patients.

## 2 MATERIALS AND METHODS

### 2.1 Study Subjects

This study was approved by the Second Affiliated Hospital of Guangxi Medical University Medicine Ethics Committee (approval number: 2020-KY-0155).

**Fig. (1):**
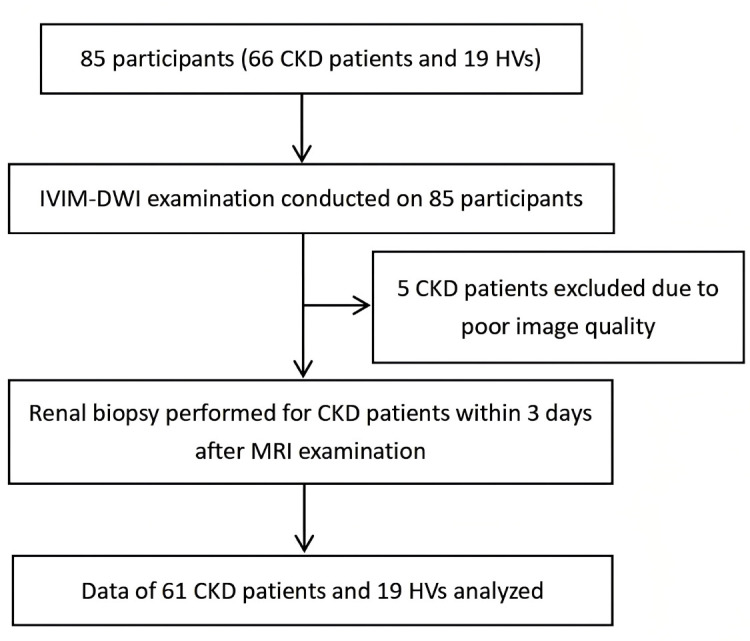
Flow chart of subject selection. **Abbreviations**: CKD: Chronic Kidney Disease; HV: Healthy Volunteer; IVIM: Intravoxel Incoherent Motion; DWI: Diffusion-Weighted Imaging; MRI: Magnetic Resonance Imaging.

The study was conducted in accordance with the Declaration of Helsinki, and informed consent was obtained from all participants. We enrolled patients with pathologically proven CKD between January 2021 and March 2023. Additionally, we enrolled healthy volunteers from the community to serve as control subjects. All participants, including CKD patients and healthy control subjects, underwent MRI examination of both kidneys, and CKD patients underwent renal puncture biopsy (Fig. **1**) in our hospital. The inclusion criteria for the CKD group were as follows: (1) CKD proven by pathological examination of a renal puncture biopsy specimen, (2) first renal puncture biopsy (*i.e*., no history of prior renal biopsy), (3) no hydronephrosis due to any reason, (4) no renal tumor, and (5) no drug treatment for 1 month prior to the MRI examination. The exclusion criteria for the CKD group were as follows: (1) polycystic kidney disease, (2) contraindication for MRI examination, and (3) image quality not meeting requirements (for example, severe respiratory artifact). The healthy volunteers had no history of renal disease and had renal function indexes within the normal reference ranges.

This study was exploratory in nature; therefore, a formal a priori sample-size calculation was not performed. The sample size was determined based on patient availability and the recruitment of all eligible CKD patients undergoing renal biopsy during the study period (**Supplementary Methods**), similar to previous feasibility studies using IVIM-MRI in renal assessment [19].

### 2.2 Clinical Evaluation

Venous blood samples were collected from all subjects prior to their bilateral renal MRI examination. The samples were used to measure the Serum Creatinine (SCr) level, and the eGFR was calculated according to the Isotope Dilution Mass Spectrometry (ID-MS)-traceable Modification of Diet in Renal Disease (MDRD) formula in eq. (**1**) [20]:

**Table:** 

eGFR = 186 × SCr^-1.154^ × (age)^-0.203^ × (0.742, if female),	(1)

Where SCr was expressed in micromoles per liter, and age was expressed in years. All CKD patients were divided into 2 groups as follows: a high eGFR group, ≥ 90 mL/min/1.73 m^2^; and a low eGFR group, < 90 mL/min/1.73 m^2^.

### 2.3 Renal Pathological Evaluation

Within 3 days after MRI, percutaneous renal biopsy was performed for all CKD patients. Biopsy was conducted on the lower pole of either kidney under B-ultrasound guidance, and 2-3 specimens (1-2 cm) were obtained. The specimens were examined using immunofluorescence, electron microscopy, and optical microscopy. A pathologist with 15 years of experience, who was blinded to the patients’ information, scored the samples for glomerular, tubulointerstitial, and vasculopathy damage using the Katafuchi semi-quantitative criteria [21]. The semi-quantitative standards are presented in Additional file 1 (Table **S1**) [22], assigned glomerular scores (0-12), tubulointerstitial scores (0-9), and vasculopathy scores (0-6).

### 2.4 MRI Examination

MRI examination was performed for all participants using a GE 3.0-T Pioneer MRI System (SIGNA Pioneer, GE Healthcare, Tokyo, Japan), with a 16-channel abdominal phased-array surface coil. All participants entered the MRI machine feet first in a supine position. The scanning scope contained both kidneys. The scanning process utilized respiratory gating technology. Single-short spin echo planar imaging was used to conduct IVIM scanning. The scanning parameters were as follows: effective repetition time; minimum exposure time; field of view = 40.0 cm × 40.0 cm; matrix = 128 × 64; slice thickness = 4.0 mm; slice gap = 0 mm; number of slices = 22; *b* values = 0, 10, 20, 30, 50, 70, 100, 150, 200, 400, 600, 800, and 1000; and number of excitations = 2, 2, 1, 1, 1, 2, 2, 4, 4, 4, 4, and 8, respectively. Spatial saturation was utilized to reduce artifacts.

### 2.5 Quantitative Parameters Derived Using the Biexponential and Stretched Exponential Models

Two radiologists with 8 and 15 years of experience were blinded to the participants’ information and trained in image post-processing and Region of Interest (ROI) measurements. IVIM data were analyzed using Functool (GE Advantage Windows v4.6) and MADC software to generate ADC, ADC_slow_, ADC_fast_, *f*, DDC, and *α* images. Diffusion parameters were derived using the following mathematical models:

#### 2.5.1 Monoexponential Model

The signal intensity S(*b*) at a given *b*-value was expressed as:

**Table:** 

S(*b*) = So· exp (-*b*· ADC),

Where So is the signal intensity without diffusion, *b* represents the diffusion-sensitizing factor, and ADC is the apparent diffusion coefficient.

#### 2.5.2 IVIM Model

Signal decay was modeled as a combination of the fast and slow diffusion components.

**Table:** 

S(*b*) = So· [ *f*· exp (-*b*· D_fast_) + (1 - *f*) · exp (-*b*· D_slow_)],

Where *f* indicates the fraction of fast diffusion, D_fast_ is the fast diffusion coefficient, and D_slow_ corresponds to the slow diffusion coefficient.

#### 2.5.3 Stretched-exponential Model

This model accounts for tissue heterogeneity by introducing a non-Gaussian diffusion term.

**Table:** 

S(*b*) = So· exp [- (*b*· DDC)”],

Where DDC represents the distributed diffusion coeffi-cient, and *α* is the anomalous exponent term, reflecting tissue complexity.

The monoexponential model was fitted using the least squares method, while the IVIM and stretched-exponential models were fitted using the Levenberg-Marquardt algorithm; these fitting approaches align with those commonly applied in prior research [23].

The images of the kidney subjected to a puncture biopsy were analyzed. The radiologists selected the largest renal hilar area in the coronal plane for ROI placement, avoiding vessels, fat, cysts, and artifacts. They placed 6-9 ROIs (35-50 mm^2^ each) on the renal cortex. The diameter of the cortical ROIs was limited to avoid exceeding the local cortical thickness, and renal margins were carefully avoided to prevent partial volume effects. The results were averaged across all ROIs. All parameter maps were synchronized to ensure anatomical consistency while minimizing measurement bias across all subjects. A schematic diagram of the parameter measurements is shown in Fig. (**2**).

### 2.6 Statistical Analysis

All data in this study were analyzed using the SPSS statistical package (SPSS, *v*27.0) and MedCalc statistical software (MedCalc, *v*20.215). The chi-square test was used to assess gender differences among the 3 study groups and differences in pathological scores between the 2 CKD groups. The Shapiro-Wilks test was used to determine whether the data were normally distributed. Data that were found to be normally distributed were expressed as mean ± standard deviation, while data with a non-normal distribution were presented as median (25% quantile, 75% quantile). The Mann-Whitney *U*-test was used to compare renal pathological scores between CKD patients with normal and abnormal renal function. Renal function indicators, ADC, and biexponential and stretched exponential model-derived parameters were compared among the control, high eGFR, and low eGFR groups by using one-way analysis of variance for data that presented a normal distribution or by using non-parametric tests (Kruskal-Wallis H test) for data that did not conform to a normal distribution. Intraclass Correlation Coefficients (ICCs) were used to evaluate interobserver consistency. Spearman correlation analysis was used to evaluate the relationship between ADC and biexponential and stretched exponential model-derived parameters of the renal cortex and renal function indexes, as well as pathological scores. Receiver Operating Characteristic (ROC) curve analysis was used to assess the diagnostic performance of ADC, biexponential and stretched exponential model-derived parameters of the renal cortex, as well as renal function indexes, among the 3 study groups. To control for multiple testing, *P*-values from the correlation and ROC analyses were adjusted using the Bonferroni method. *P* < 0.05 was deemed to indicate statistical significance.

## 3 RESULTS

### 3.1 General Characteristics

In total, 85 subjects, consisting of 66 CKD patients and 19 healthy volunteers, participated in this prospective study. After excluding 5 CKD patients with poor image quality, we ultimately analyzed the data of 61 CKD patients, including 37 men and 24 women, with an age of 31.80 ± 12.53 years. The control group consisted of 7 men and 12 women, with an age of 26.95 ± 10.29 years. Among the CKD patients, 33 were assigned to the high eGFR group (≥ 90 mL/min/1.73 m^2^), and 28 were included in the low eGFR group (< 90 mL/min/1.73 m^2^). No significant gender differences were found among the high eGFR, low eGFR, and control groups (*P* > 0.05). The following pathological subtypes of CKD were detected among the patients: IgA nephropathy, 30 patients; membranous nephropathy, 13 patients; minimal change disease, 11 patients; focal segmental glomerulosclerosis, 5 patients; and mesangial proliferative glomerulonephritis, 2 patients. The general characteristics of the participants are listed in Table **1**.

### 3.2 Comparison of Quantitative Parameters Derived Using the Biexponential and Stretched Exponential Models Among the Study Groups

The quantitative parameters derived using the biexponential and stretched exponential models for the renal cortex in the 3 study groups are listed in Table **2**. ADC_slow_, ADC_fast_, *f*, DDC, and *α* values showed a decreasing trend across the control, high eGFR, and low eGFR groups. The ADC, ADC_slow_, and DDC values differed significantly between the control and low eGFR groups, as well as between the high and low eGFR groups (*P* < 0.05), but not between the control and high eGFR groups (all *P* > 0.05). The ADC_fast,_ *f*, and *α* values differed significantly between the control, high eGFR, and low eGFR groups, in pairwise comparisons (all *P* < 0.05).

ADC, biexponential and stretched exponential model-derived parameters of the renal cortex, as measured by the 2 radiologists, along with their ICCs and 95% confidence intervals, are shown in Additional file 1 (Table **S2**). Analysis of inter-observer reproducibility showed excellent agreement for the ADC, ADC_fast_, *f*, DDC, and *α* values, with ICCs ranging from 0.949 to 0.973. For ADC_slow_ values, substantial agreement was reached between the 2 observers (ICC: 0.722). Therefore, measurements from a single radiologist were randomly selected for further statistical analysis.

### 3.3 Comparison of Renal Pathological Scores between the High and Low eGFR Groups

Comparison of the renal pathological scores between CKD patients with high and low eGFR (Table **3**) revealed that the glomerular score, tubulointerstitial score, vasculopathy score, and total pathological score were all significantly higher in the low eGFR group than in the high eGFR group (*P* < 0.05).

### 3.4 Correlation of Quantitative Parameters Derived Using the Biexponential and Stretched Exponential Models with eGFR and Pathological Scores

The correlation of quantitative parameters derived using the biexponential and stretched exponential models with eGFR and pathological renal scores is presented in Table **4**. ADC ADC_fast_, *f* and stretched exponential model-derived parameters in the renal cortex were positively correlated with eGFR, among which ADC, ADC_fast_, *f*, and DDC were strongly correlated with eGFR (*r* = 0.640, 0.668, 0.609, and 0.667, respectively; all *P* < 0.05). ADC_fast_, *f*, and *α* were negatively correlated with the glomerular score, tubulointerstitial score, vasculopathy score, and total renal score, among which cortical ADC_fast_ and *f* were strongly correlated with the tubulointerstitial score (*r* = -0.621 and -0.604, respectively; both *P* < 0.05) and the total score (*r* = -0.627 and -0.620, respectively; both *P* < 0.05). The MRI maps and corresponding pathological changes in the 2 CKD groups are shown in Figs. (**3** and **4**).

### 3.5 Diagnostic Performance of Quantitative Parameters Derived Using the Biexponential and Stretched Exponential Models

**Fig. (5):**
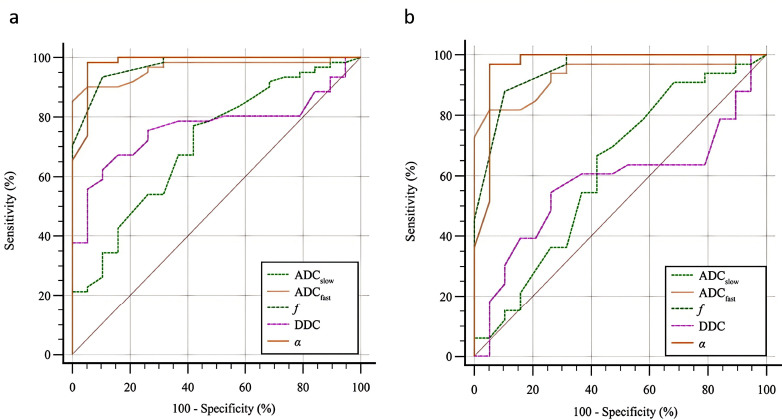
ROC curves of quantitative parameters derived using the biexponential and stretched exponential models for detecting CKD. **(a)** ROC curve analysis for CKD (versus the control group) showed that the AUC values of ADC_slow_, ADC_fast_, *f*, DDC, and *α* were 0.711, 0.964, 0.974, 0.766, and 0.981, respectively (all *P* < 0.05). **(b)** ROC curve analysis for high eGFR (versus the control group) showed that the AUC values of ADC_slow_, ADC_fast_, *f*, DDC, and *α* were 0.622 (*P >* 0.05), 0.933 (*P* < 0.05), 0.952 (*P* < 0.05), 0.576 (*P >* 0.05), and 0.966 (*P* < 0.05), respectively. **Abbreviations**: CKD: Chronic Kidney Disease; ROC: Receiver Operating Characteristic; AUC: Area Under the Curve; eGFR: estimated Glomerular Filtration Rate; ADC_slow_: True Diffusion Coefficient; ADC_fast_: Pseudo Diffusion Coefficient; *f*: Perfusion Fraction; DDC: Distributed Diffusion Coefficient; *α*: Alpha.

ROC curve analysis revealed that the Areas Under the Curve (AUCs) for the *α*, *f*, and ADC_fast_ values for the diagnosis of CKD were 0.981, 0.974, and 0.964, respectively. The corresponding values for DDC and ADC_slow_ were lower, at 0.766 and 0.711, respectively, while the AUC of ADC showed no significant diagnostic efficacy in the CKD group (*P >* 0.05). The AUCs of the ADC_fast_, *f*, and *α* values for the diagnosis of CKD with high eGFR (≥ 90 mL/min/1.73 m^2^) were 0.933, 0.952, and 0.966, respectively, while the ADC, ADC_slow_, and DDC values showed no prominent diagnostic efficacy in the high eGFR group (*P >* 0.05) (Table **5** and Fig. **5**).

## 4 DISCUSSION

This study showed that the quantitative parameters derived using the biexponential and stretched exponential models, namely, ADC_slow_, ADC_fast_, *f*, DDC, and *α*, displayed a decreasing trend across the control, high eGFR, and low eGFR groups. ADC, ADC_fast_, *f*, and DDC values showed a significant positive correlation with eGFR (*r* = 0.640, 0.668, 0.609, and 0.667, respectively; *P* < 0.05), and ADC_fast_ and *f* values showed a significant negative correlation with tubulointerstitial score (*r* = -0.621 and -0.604, respectively; *P* < 0.05). The *α*, *f*, and ADC_fast_ values demonstrated a relatively high diagnostic accuracy in differentiating control subjects from CKD patients, as well as in differentiating the control subjects from CKD patients with a high eGFR (AUC > 0.9, *P* < 0.05 for all).

The biexponential model yields diffusion and perfusion data (ADC_slow_, ADC_fast_, *f*); the stretched exponential model captures microstructural complexity and diffusion heterogeneity (DDC, α). In theory, the use of the biexponential and stretched exponential models allowed us to consider the perfusion characteristics of the microcirculation, the realistic diffusion of water molecules, and all protons in cells within each voxel, and more accurately and genuinely reflect the renal dysfunction and pathological changes in CKD patients.

In the present study, patients were grouped according to their eGFR, as calculated using the 2006 Chinese modification of the ID-MS MDRD formula [20]. The eGFR significantly differed between the control group and the low eGFR group, and between the high and low eGFR groups (both *P* < 0.05); however, no significant difference in eGFR was found between the control and high eGFR groups (*P >* 0.05), which indicates that eGFR is of limited value in assessing early renal damage in CKD patients [24, 25].

Different subtypes of CKD share common pathological mechanisms, including glomerulosclerosis, tubular atrophy, and renal interstitial fibrosis, all of which ultimately progress to renal fibrosis [26]. During the progression of renal fibrosis, the renal tubular cell area correlates closely with eGFR, potentially due to non-functional glomeruli resulting from tubular atrophy, which causes a decline in filtration. Myofibroblast and inflammatory cell deposition increase cell density, thereby reducing extracellular water and limiting the diffusion of water molecules. Mao *et al*. demonstrated that renal cortical perfusion is associated with peritubular capillary density, which is compromised by CKD-induced pathologies, such as tubular atrophy, glomerulosclerosis, and arteriolar wall thickening, leading to decreased perfusion [27]. Moreover, with the progression of CKD, the increasing renal fibrosis and deposition of extracellular components further limit water molecule diffusion and reduce renal cortical perfusion [28].

Research has indicated that biexponential parameters exhibit inconsistencies with conclusions regarding CKD staging and renal histology, which may be attributed to their inherent limitations in the IVIM model, sensitivity to noise, and the complexity of renal physiology [23, 29]. The number and distribution of b-values have a decisive impact on the estimation of D_slow_ and D_fast_. In our study, the number and proportion of b-values ≤ 200 s/mm^2^ were increased in the IVIM scan parameters. Subjects underwent breathing training prior to the scan, and respiratory gating technology was used during the scan. These changes may have a positive effect on the stability and repeatability of the biexponential parameters.

This study revealed that pathological scores and total renal scores differed significantly between the high and low eGFR groups (*P* ≤ 0.002), and showed that renal cortex ADC_fast_, *f*, and *α* were negatively correlated with glomerular, tubulointerstitial, vasculopathy, and total renal scores. These results indicate that the above parameters can effectively evaluate renal pathology in CKD. Notably, the ADC_fast_ and *f* values correlated significantly with the tubulointerstitial score (*r* = -0.621 and -0.604, respectively; both *P* < 0.05) and total renal score (*r* = -0.627 and -0.620, respectively; both *P* < 0.05), suggesting that ADC_fast_ and *f* values independently indicate tubulointerstitial damage.

The limitation of water-molecule diffusion and the reduction of tissue perfusion lead to a decline in the ADC value of the renal parenchyma [30]. Çakmak *et al*. and Yalçin-Şafak *et al*. found that in CKD patients, the ADC value of the renal parenchyma declines with a decline in eGFR, indicating a positive correlation with eGFR and a relationship with the clinical phase of CKD; however, the ADC value was found not to differ between control subjects and CKD patients with a high eGFR (≥ 90 mL/min/1.73 m^2^) [31, 32]. Our results are consistent with the above observations, which confirm that renal cortical ADC values can reflect the extent of renal dysfunction in CKD, but do not indicate early renal damage in CKD patients.

In our study, the ADC_slow_, ADC_fast_, and *f* values of the renal cortex showed a declining trend across the control, high eGFR, and low eGFR groups; moreover, ADC_fast_ and *f* values showed significant differences among the 3 groups, but ADC_slow_ values did not differ between the control and high eGFR groups. The ADC_fast_ and *f* values were positively correlated with eGFR (*r* = 0.668 and 0.609, respectively). As stated earlier, the pathological changes of CKD limit the diffusion of water molecules and reduce perfusion of the microcirculation, leading to decreased renal cortical ADC_slow_, ADC_fast_, and *f* values with advancing renal fibrosis. While ADC_slow_ values were similar between the control and high eGFR groups, ADC_fast_ and *f* values varied significantly, indicating their ability to detect early renal damage in CKD. This implies that early in CKD, microcirculatory perfusion changes in the renal cortex may precede alterations in actual water molecule diffusion. The sensitivity of ADC_fast_ and *f* to early CKD damage may relate to the inclusion of multiple low b-values in IVIM imaging, enhancing the impact of microcirculation perfusion on ADC_fast_ values [33].

The DDC value represents the average diffusion rate within a voxel, while the *α* value represents the heterogeneity of diffusion. Both parameters reflect the heterogeneity and complexity of tissue microstructure and are commonly used for the evaluation of neoplastic lesions [17, 34, 35]. In our study, the DDC and *α* values of the renal cortex obtained using the stretched exponential model showed a declining trend across the control, high eGFR, and low eGFR groups. Pairwise comparisons of *α* values revealed significant differences among the 3 groups (all *P* < 0.05), but DDC values did not differ between the control and high eGFR groups. This suggests that *α* values can detect CKD-related renal damage earlier than DDC values. Both DDC and *α* values were positively correlated with eGFR (*r* = 0.667 and 0.525, respectively), and negatively correlated with glomerular score (*r* = -0.531 and -0.453, respectively) and tubulointerstitial score (*r* = -0.563 and -0.506, respectively). This indicates that DDC and *α* values of the renal cortex can reflect glomerular filtration function and the extent of pathological lesions in CKD. During the progression of CKD, pathological changes such as increased glomerulosclerosis, interstitial fibrosis, and increased cell density result in the reduction of the extracellular space and damage to the peritubular capillaries, which in turn lead to an increase in the complexity of the tissue structure and affect the rate of water-molecule diffusion within voxels, thereby altering the DDC and *α* values.

Overall, the *α*, *f*, and ADC_fast_ values of the renal cortex showed relatively high diagnostic accuracy in differentiating control subjects from CKD patients (AUC = 0.981, 0.974, and 0.960, respectively) as well as in differentiating control subjects from CKD patients with a high eGFR (AUC = 0.966, 0.952, and 0.933, respectively). This indicates that the *α*, *f*, and ADC_fast_ values of the renal cortex have substantial clinical potential in detecting CKD and CKD-related early renal damage, and may be superior to ADC, other parameters derived using biexponential and stretched exponential models, and eGFR for this purpose.

There is an age difference between the control group and the experimental group in this experiment. However, a previous study has indicated that the cytological differences in the kidneys of healthy individuals aged 20-30 and 30-40 years are not statistically significant [36].

Although IVIM-derived parameters show promise, their clinical implementation requires standardized acquisition and analysis protocols across vendors and field strengths. Parameter values are sensitive to b-value distribution, signal-to-noise ratios, and fitting algorithms, which may vary among scanners. Establishing harmonized protocols and conducting multicenter reproducibility studies will be essential before IVIM metrics can be adopted for the individualized monitoring of CKD. The increasing complexity of quantitative MRI data also raises issues of data management and patient privacy. Multi-institutional collaborations necessitate secure data-sharing frameworks compliant with data-protection regulations. Recent developments in federated learning and privacy-preserving analytics may enable cross-center model validation without compromising patient confidentiality [37].

## 5 LIMITATIONS

This study has certain limitations. First, for the consistency of the quantitative parameter set for MRI, this prospective study was designed to be conducted in a single center. Future multicenter studies, incorporating a larger sample size and including more CKD subtypes, can enhance the generalizability of the research results. Second, all MRI examinations were performed on a single 3.0-T scanner using a uniform acquisition protocol; thus, our findings may not be directly generalizable to other platforms or 1.5-T scanners. Third, reproducibility testing through repeated scans in the same individuals was not conducted, and the inter-session variability of quantitative parameters remains to be clarified. Fourth, due to the unclear demarcation between the renal cortex and medulla on IVIM images, this experiment only measured data from the renal cortex. Future studies will aim to utilize machine learning-based automated ROI placement and segmentation techniques in research. Finally, there was a spatial mismatch between the biopsy site and the MRI-derived ROIs. While biopsies were performed in the lower pole for safety, ROIs were distributed across the entire renal cortex. This discrepancy may partly explain the moderate correlations observed between the imaging parameters and pathological scores. Future studies integrating image-guided biopsies and histopathological analysis could provide more spatially matched validation.

## CONCLUSION

In conclusion, this study revealed that ADC_fast_, *f*, and *α* values of the renal cortex could detect early renal damage in CKD patients with an eGFR ≥ 90 mL/min/1.73 m^2^. Moreover, the ADC_fast_ and *f* values of the renal cortex were significantly correlated with tubulointerstitial damage. Thus, parameters derived using the biexponential and stretched exponential models have the potential for clinical application in the individualized monitoring and follow-up of CKD patients.

## Figures and Tables

**Fig. (2):**
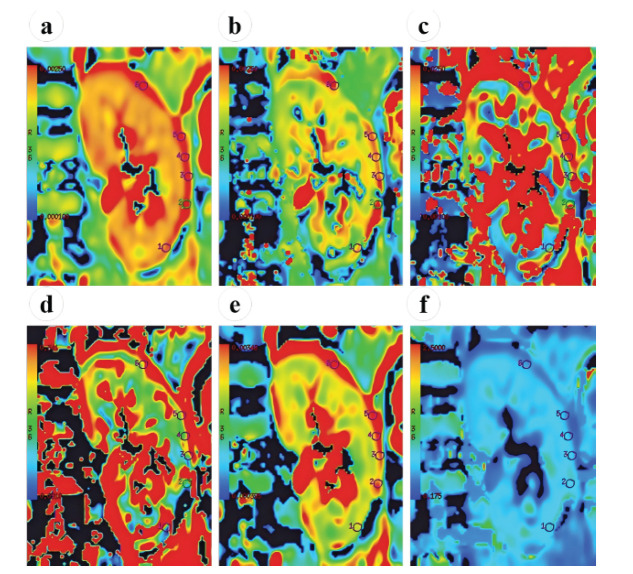
An example of ROI delineation in a section passing through the hilum of the right kidney. Six ROIs measuring 45 mm^2^ each were manually delineated in the renal cortex on the **(a)** ADC map. Then, the ROIs were copied to the **(b)** ADC_slow_ map, **(c)** ADC_fast_ map, **(d)** *f* map, **(e)** DDC map, and **(f)** *α* map. **Abbreviations**: ROI: Region of Interest; ADC: Apparent Diffusion Coefficient; ADC_slow_: true Diffusion Coefficient; ADC_fast_: Pseudo Diffusion Coefficient; *f:* Perfusion Fraction; DDC: Distributed Diffusion Coefficient; *a*: Alpha.

**Fig. (3):**
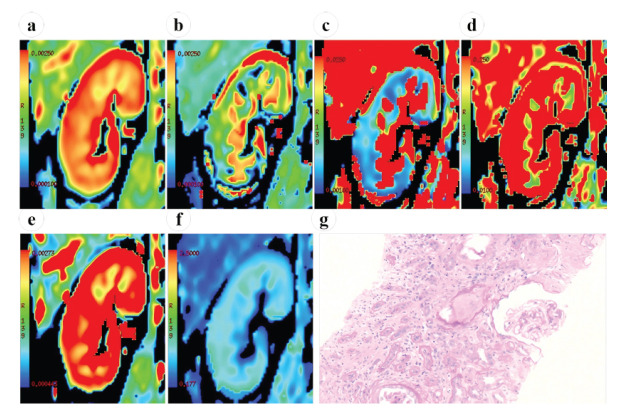
Magnetic resonance images in the coronal plane and corresponding pathological changes in a 45-year-old man with CKD and a low eGFR (66.3 mL/min/1.73 m^2^). **(a)** ADC map (ADC: 2.15 × 10^-3^ mm^2^/s). **(b)** ADC_slow_ map (ADC_slow_: 1.65 × 10^-3^ mm^2^/s). **(c)** ADC_fast_ map (ADC_fast_: 15.6 × 10^-3^mm^2^/s). **(d)** *f* map (*f*: 0.19). **(e)** DDC map (DDC: 2.28 × 10^-3^ mm^2^/s). **(f)** *α* map (*α*: 0.55). **(g)** Mild glomerular mesangial cell and stromal hyperplasia, vacuolar degeneration of the renal tubular epithelial cells, a small amount of inflammatory-cell infiltration and fibrosis in the renal interstitium, arteriole-wall thickening, arteriole hyalinization, and lumen stenosis are observed (periodic acid-Schiff staining; magnification, ×20). The glomerular score is 2; the tubulointerstitial score is 6; and the vasculopathy score is 2. **Abbreviations**: CKD: Chronic Kidney Disease; eGFR: estimated Glomerular Filtration Rate; ADC: Apparent Diffusion Coefficient; ADC_slow_: True Diffusion Coefficient; ADC_fast_: Pseudo Diffusion Coefficient; *f*: Perfusion Fraction; DDC: Distributed Diffusion Coefficient; *α*: Alpha.

**Fig. (4):**
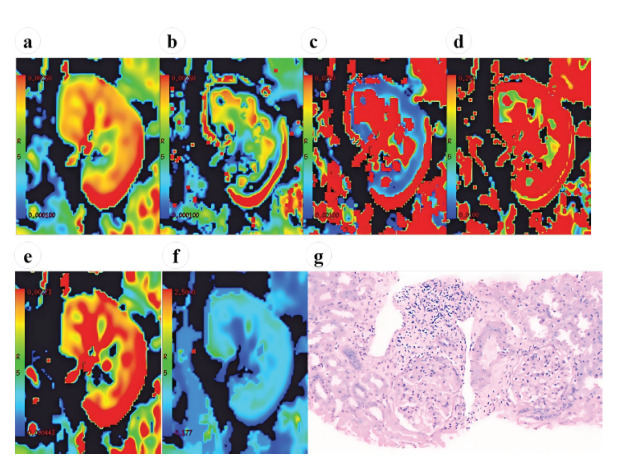
Magnetic resonance images in the coronal plane and corresponding pathological changes in a 43-year-old woman with CKD and a high eGFR (95 mL/min/1.73 m^2^). (**a**) ADC map (ADC: 2.33 × 10^-3^ mm^2^/s). (**b**) ADC_slow_ map (ADC_slow_: 1.75 × 10^-3^ mm^2^/s). (**c**) ADC_fast_ map (ADC_fast_: 26.5 × 10^-3^ mm^2^/s). (**d**) *f* map (*f*: 0.29). (**e**) DDC map (DDC: 3.12 × 10^-3^ mm^2^/s). (**f**) *α* map (*α*: 0.64). (**g**) Mild hyperplasia of the glomerular mesangial cells and stroma, granular and vacuolar degeneration of the renal tubular epithelial cells, focal inflammatory-cell infiltration of the renal interstitium, fibrosis, arteriole-wall thickening, and lumen stenosis are seen (periodic acid-Schiff staining; magnification, ×20). The glomerular score is 2; the tubulointerstitial score is 3; and the vasculopathy score is 1. **Abbreviations**: CKD: Chronic Kidney Disease; eGFR: estimated Glomerular Filtration Rate; ADC: Apparent Diffusion Coefficient; ADC_slow_: True Diffusion Coefficient; ADC_fast_: Pseudo Diffusion Coefficient; *f*: Perfusion Fraction; DDC: Distributed Diffusion Coefficient; *α*: Alpha.

**Table 1:** General characteristics of the study participants.

**Parameter**	**Control Group** **(n = 19)**	**High eGFR Group** **(n = 33)**	**Low eGFR Group** **(n = 28)**	***P-*value**
**Age (Years)**	26.95 ± 10.29	28.85 ± 11.33	35.29 ± 13.19	0.038
**Gender**	-	-	-	0.057
Male	7	17	20	-
Female	12	16	8	-
Creatinine (μmol/L)	59.00 (56.00, 67.00)	63.00 (55.00, 73.50)	114.50 (94.50, 137.75)	1^a^, <0.001^b,c^
eGFR (mL/min/1.73 m^2^)	122.39 (98.45, 128.84)	116.63 (101.63, 150.55)	62.51 (53.03, 78.66)	1^a^, <0.001^b,c^
**Pathological Subtype**		-	-	0.240
IgA nephropathy	-	14 (22.9%)	16 (26.2%)	-
MN	-	9 (14.8%)	4 (6.6%)	-
MCD	-	7 (11.5%)	4 (6.6%)	-
FSGS	-	3 (4.9%)	2 (3.3%)	-
MPGN	-	0 (0.0%)	2 (3.3%)	-

eGFR: estimated Glomerular Filtration Rate; IgA: Immunoglobulin A; MN: Membranous Nephropathy; MCD: Minimal Change Disease; FSGS: Focal Segmental Glomerulosclerosis; MPGN: Mesangial Proliferative Glomerulonephritis a, represents the difference between the control group and the high eGFR (≥ 90 mL/min/1.73 m^2^) group. b, represents the difference between the control group and the low eGFR (< 90 mL/min/1.73 m^2^) group. c, represents the difference between the high and low eGFR groups.

**Table 2:** Comparison of quantitative parameters derived using the biexponential and stretched exponential models among the control, high eGFR, and low eGFR groups.

**Parameter**	**Control Group**	**High eGFR Group**	**Low eGFR Group**	***P* ^1^**	***P* ^2^**	***P* ^3^**
ADC (×10^-3^ mm^2^/s)	2.33 (2.25, 2.40)	2.40 (2.31, 2.51)	2.03 (1.80, 2.18)	0.878	<0.001	0.003
ADC_slow_ (×10^-3^ mm^2^/s)	1.80 (1.70, 1.93)	1.73 (1.70, 1.80)	1.63 (1.50, 1.72)	0.700	<0.001	<0.001
ADC_fast_ (×10^-3^ mm^2^/s)	31.40 (30.00, 32.25)	26.75 (25.00, 28.75)	14.5 (10.58, 16.69)	0.020	<0.001	<0.001
*f* (%)	0.34 ± 0.04	0.28 ± 0.02	0.17 ± 0.06	<0.001^a^	<0.001^a^	<0.001^a^
DDC (×10^-3^ mm^2^/s)	3.30 (3.20, 3.35)	3.23 (3.01, 3.38)	2.24 (2.13, 2.41)	1.000	<0.001	<0.001
*α* (%)	0.75 (0.72, 0.79)	0.63 (0.61, 0.66)	0.54 (0.51, 0.56)	0.001	<0.001	<0.001

eGFR: estimated Glomerular Filtration Rate; ADC: Apparent Diffusion Coefficient; ADC_slow_: True Diffusion Coefficient; ADC_fast_: Pseudo Diffusion Coefficient; *f*: Perfusion Fraction; DDC: Distributed Diffusion Coefficient; *α*: Alpha *P*^1^, represents the difference between the control and high eGFR (≥ 90 mL/min/1.73 m^2^) group. *P*^2^, represents the difference between the control and low eGFR (< 90 mL/min/1.73 m^2^) group. *P*^3^, represents the difference between the high and low eGFR groups. a, indicates parameters evaluated using analysis of variance; the remaining parameters were evaluated using the Kruskal-Wallis H test.

**Table 3:** Comparison of pathological scores between the high and low eGFR groups.

**Score**	**High eGFR Group**	**Low eGFR Group**	***P* value**
Glomerular	2 (1, 3)	5 (2.25, 6.75)	<0.001
Tubulointerstitial	2 (1, 3)	5 (2.25, 6.75)	<0.001
Vasculopathy	0 (0, 1)	2 (0.25, 2)	0.002
Total	5 (2, 8)	11 (7.25, 15.75)	<0.001

eGFR: estimated Glomerular Filtration Rate.

**Table 4:** Correlation of quantitative parameters derived using the biexponential and stretched exponential models with eGFR and pathological scores.

**Parameter**	**eGFR**		**Glomerular Score**		**Tubulointerstitial Score**		**Vasculopathy Score**		**Total Score**	
*r*	*P^'^*	*r*	*P^'^*	*r*	*P^'^*	*r*	*P^'^*	*r*	*P^'^*
ADC (×10^-3^ mm^2^/s)	0.640	0.000	-0.478	0.000	-0.582	0.000	-0.351	0.150	-0.563	0.000
ADC_slow_ (×10^-3^ mm^2^/s)	0.285	0.300	-0.417	0.030	-0.412	0.030	-0.194	1.000	-0.423	0.030
ADC_fast_ (×10^-3^ mm^2^/s)	0.668	0.000	-0.536	0.000	-0.621	0.000	-0.442	0.000	-0.627	0.000
*f* (%)	0.609	0.000	-0.542	0.000	-0.604	0.000	-0.430	0.030	-0.620	0.000
DDC (×10^-3^ mm^2^/s)	0.667	0.000	-0.531	0.000	-0.563	0.000	-0.356	0.150	-0.580	0.000
*α* (%)	0.525	0.000	-0.453	0.000	-0.506	0.000	-0.436	0.000	-0.534	0.000

*P^'^*were adjusted using the Bonferroni method. eGFR: estimated Glomerular Filtration Rate; ADC: Apparent Diffusion Coefficient; ADC_slow_: True Diffusion Coefficient; ADC_fast_: Pseudo Diffusion Coefficient; *f*: Perfusion Fraction; DDC: Distributed Diffusion Coefficient; *α*: Alpha.

**Table 5:** Diagnostic performance of quantitative parameters derived using the biexponential and stretched exponential models in detecting CKD and high eGFR with the Control group.

**Parameter**	**AUC**	**95% CI**	**Youden index**	**Threshold**	**Sensitivity**	**Specificity**	***P^'^***
^a^ADC (× 10^-3^ mm^2^/s)	0.638	0.523-0.743	0.344	≤2.13	34.43%	100.00%	0.168
^a^ADC_slow_ (× 10^-3^ mm^2^/s)	0.711	0.599-0.807	0.349	≤1.75	77.05%	57.89%	0.012
^a^ADC_fast_ (× 10^-3^ mm^2^/s)	0.964	0.896-0.993	0.853	≤28.00	85.25%	100.00%	0.000
^a^*f* (%)	0.974	0.911-0.997	0.829	≤0.30	93.44%	89.47%	0.000
^a^DDC (× 10^-3^ mm^2^/s)	0.766	0.658-0.854	0.518	≤3.10	62.30%	89.47%	0.000
^a^*α* (%)	0.981	0.923-0.999	0.931	≤0.69	98.36%	94.74%	0.000
^b^ADC (× 10^-3^ mm^2^/s)	0.621	0.476-0.752	0.231	>2.30	75.76%	47.37%	0.876
^b^ADC_slow_ (× 10^-3^ mm^2^/s)	0.622	0.477-0.753	0.270	≤1.85	84.85%	42.11%	0.924
^b^ADC_fast_ (× 10^-3^ mm^2^/s)	0.933	0.828-0.984	0.766	≤28.75	81.82%	94.74%	0.000
^b^*f* (%)	0.952	0.854-0.992	0.774	≤0.30	87.88%	89.47%	0.000
^b^DDC (× 10^-3^ mm^2^/s)	0.576	0.431-0.712	0.282	≤3.22	54.55%	73.68%	1.000
^b^*α* (%)	0.966	0.874-0.997	0.917	≤0.69	96.97%	94.74%	0.000

*P^'^*were adjusted using the Bonferroni method. CKD: Chronic Kidney Disease; eGFR: estimated Glomerular Filtration Rate; AUC: Area Under the Curve; CI: Confidence Interval; ADC: Apparent Diffusion Coefficient; ADC_slow_: True Diffusion Coefficient; ADC_fast_: Pseudo Diffusion Coefficient; *f*: perfusion Fraction; DDC: Distributed Diffusion Coefficient; *α*: Alpha. a, Control group (n = 19) *vs*. CKD group (n = 61). b, Control group (n = 19) *vs*. high eGFR (≥ 90 mL/min/1.73 m^2^) group (n = 33).

## Data Availability

The data of current study are available from corresponding author, [W.Y], on a reasonable request.

## LIST OF ABBREVIATIONS

IVIM: Intravoxel Incoherent Motion
CKD: Chronic Kidney Disease
ADC: Apparent Diffusion Coefficient
ADC_slow_: True Diffusion Coefficient
ADC_fast_: Pseudo-Diffusion Coefficient
*f*: Perfusion Fraction
DDC: Distributed Diffusion Coefficient
*α*: Alpha
eGFR: Estimated Glomerular Filtration Rate
AUC: Area Under the Curve
KDIGO: Kidney Disease: Improving Global Outcomes
MRI: Magnetic Resonance Imaging
IgA: Immunoglobulin A
SCr: Serum Creatinine
ID-MS: Isotope Dilution Mass Spectrometry
MDRD: Modification of Diet in Renal Disease
ROI: Region of Interest
ICCs: Intraclass Correlation Coefficients
ROC: Receiver Operating Characteristic

## STANDARDS OF REPORTING

STROBE guidelines were followed.
