# Supplementary material for: Quantitative Parameters Derived Using the Biexponential and Stretched Exponential Models for the Detection of Early Renal Impairment in Chronic Kidney Disease
Source: Curr Med Imaging. 2025 Nov 24;21:e15734056445507. doi: 10.2174/0115734056445507251111061849 (PMC13223502; doi:10.2174/0115734056445507251111061849)
Supplement: Supplementary file 1 [file CMIM-21-E15734056445507_SD1.pdf]

1573-4056/25 *Send Orders for Reprints to reprints@benthamscience.net*

BENTHAM SCIENCE
CrossMark

Current Medical Imaging

Content list available at: https://benthamscience.com/journals/cmir

Supplementary Material

Quantitative Parameters Derived Using the Biexponential and Stretched Exponential Models for the Detection of Early Renal Impairment in Chronic Kidney Disease

Yi Dai[1,2,#], Zhucheng Lu[2,3,#], Yidi Chen[1], Keqiang Huang[4], Zhenyuan Xia[2], Lan Lan[2], Wei Li[5], Haiyuan Wei[5], Xuejie Yang[6], Xiamei Chen[7], Liling Long[1] and Wenzhao Yuan[1,*]

[1]*Department of Radiology, The First Affiliated Hospital of Guangxi Medical University, No. 6 Shuangyong Road, Nanning, Guangxi - 530021, China*
[2]*Department of Radiology, The Second Affiliated Hospital of Guangxi Medical University, No. 166 Daxuedong Road, Nanning, Guangxi - 530007, China*
[3]*Department of Radiology, Liuzhou Traditional Chinese Medical Hospital, No. 32 Jiefang North Road, Liuzhou, Guangxi - 545001, China*
[4]*Department of Pathology, The First Affiliated Hospital of Guangxi University of Chinese Medicine, No. 89-9 Dongge Road, Nanning, Guangxi - 530023, China*
[5]*Department of Nephrology, The Second Affiliated Hospital of Guangxi Medical University, No. 166 Daxuedong Road, Nanning, Guangxi - 530007, China*
[6]*Guangxi University of Chinese Medicine, No. 13 Wuhe Avenue, Nanning, Guangxi - 530200, China*
[7]*MR Application, GE Healthcare Co., Ltd., Beijing - 100176, China*

SUPPLEMENTARY METHODS

This study was prospectively designed as an exploratory diagnostic-ccuracy/feasibility investigation focusing on IVIM-derived parameters. A formal a priori sample-size calculation was not performed at the time of design due to the pilot nature and the constraint of enrolling consecutive biopsy-proven CKD patients within the recruitment window. To address the reviewer's concern, we now provide a brief sample-size rationale aligned with the study objectives and add a Supplementary Methods subsection with standard formulas commonly used for observational diagnostic-accuracy studies (correlations, ROC AUC, and precision of sensitivity/specificity).

In brief, our analytic goals were: 1) to estimate correlations between MRI parameters and eGFR/pathology; and 2) to evaluate discriminative performance (ROC AUC) for differentiating CKD (and early CKD with preserved eGFR) from controls.

Using conventional Fisher-z and Hanley–McNeil approximations, the available sample (CKD n=61; controls n=19) is adequate to detect moderate-to-large effects at $\alpha=0.05$ with high power. Specifically, with n=61 CKD patients, the study has >80% power to detect $|\rho| \approx 0.33–0.35$ (two-sided) in correlation analyses; and with 61 cases/19 controls, the study has >80% power to detect AUC $\geq$ 0.75 against the null AUC=0.5. Our observed effects (*e.g.*, Spearman $|r|\approx0.60–0.67$ and AUC≈0.80–0.95 for α, f, ADCfast) exceed those design-agnostic thresholds, supporting the adequacy of the enrolled sample for an exploratory study. We now clearly state these points in the manuscript and include formulae for transparency.

Table S1. Semiquantitative standards for glomerulus, tubulointerstitial and vasculopathy scoring.

Score	Glomerulus Lesion Score (%)			Tubulointerstitial Lesion Score (%)			Vasculopathy Score (%)	
	Glomeruluscell Proliferation Rate	Segmental Lesions	Glomerulosclerosis	Interstitial Fibrosis	Tubular Atrophy	Interstitial Inflammatory Infiltration	Vessel Wall Thickening	Hyaline Degeneration
1	≤25	≤10	≤10	≤25	≤25	≤25	≤10	≤25
2	25-50	10-25	10-25	25-50	20-50	20-50	10-25	20-50
3	50-75	25-50	25-50	≥50	≥50	≥50	≥25	≥50
4	≥75	≥50	≥50	NA	NA	NA	NA	NA

Abbreviations: NA = Not Applicable

DOI: 10.2174/0115734056445507251111061849, 2025, *21*, e15734056445507

Table S2. Inter-Observer reproducibility for the measurements of quantitative parameters derived using the biexponential and stretched exponential models.

Parameter	ICC	95%CI
ADC ($\times 10^{-3} mm^2/s$)	0.958	0.936-0.973
ADC_{slow} ($\times 10^{-3} mm^2/s$)	0.722	0.598-0.812
ADC_{fast} ($\times 10^{-3} mm^2/s$)	0.972	0.957-0.982
f (%)	0.964	0.945-0.972
DDC ($\times 10^{-3} mm^2/s$)	0.949	0.922-0.967
α (%)	0.973	0.957-0.982

© 2025 The Author(s). Published by Bentham Science Publisher.

This is an open access article distributed under the terms of the Creative Commons Attribution 4.0 International Public License (CC-BY 4.0), a copy of which is available at: https://creativecommons.org/licenses/by/4.0/legalcode. This license permits unrestricted use, distribution, and reproduction in any medium, provided the original author and source are credited.
